# 21771 HIV Prevention among HIV-Negative Latino Males: Identifying Sociocultural Factors Associated with Pre-exposure Prophylaxis

**DOI:** 10.1017/cts.2021.615

**Published:** 2021-03-30

**Authors:** Juan Pablo Zapata, Ed de St. Aubin

**Affiliations:** Marquette University

## Abstract

ABSTRACT IMPACT: The broad goal of this investigation is to inform the development of culturally sensitive HIV prevention strategies to reduce specific challenges pertaining to PrEP uptake and utilization for Latino men. OBJECTIVES/GOALS: HIV is a significant public health concern affecting Latinos in the U.S. Daily use of pre-exposure prophylaxis (PrEP) effectively prevents HIV infection and has the potential to curb HIV epidemics. The objective of this study is to examine how sociocultural variables impact PrEP-related services among HIV-negative Latinxs. METHODS/STUDY POPULATION: The current study is a mixed-method investigation. Participants will include Latinx adult patients seeking services at an HIV community clinic. Approximately 150 participants will be recruited for the study. Participants who are eligible will complete sociocultural, mental health and PrEP-related measures. For the applied aim, community stakeholders will be recruited who serve the Latinx community. Upon completion of data collection, the data analytic plan is as follows: Aim 1, to establish the relationship between each sociocultural variable and PrEP uptake/utilization, preliminary analyses (i.e., correlations and regression analyses considering co-variates) will be conducted. Aim 2, grounded theory techniques will be conducted to establish community-informed practices to increase the use of PrEP. RESULTS/ANTICIPATED RESULTS: Relatively little is known about cultural factors that may impede PrEP uptake among Latinx MSM. Several researchers have identified specific factors such as language, acculturation, familismo, and similar cultural norms as significant barriers to care (Page et al., 2017). It is expected that each of these variables will contribute significant variance to willingness to use PrEP. Specifically, negative relationships are expected between fatalism and machismo and lower stages on the PrEP Contemplation Ladder. Comparably, a negative relationship is expected between the Hispanic acculturation subscale and lower stages on the PrEP Contemplation Ladder. It is however, hypothesized that there will be a positive relationship between familism and the non-Hispanic acculturation sub-scale. DISCUSSION/SIGNIFICANCE OF FINDINGS: Despite important advances in health to prevent HIV infection, HIV rates among Latinx MSM continue to rise. This investigation will have the potential to inform the development of culturally sensitive prevention strategies. By collecting qualitative data from key community stakeholders, this project will also directly inform a CBPR prevention.

